# EFFECTS OF SOCIAL SUPPORT ON PERSONS’ NEUROLOGICAL WELL-BEING:IMPLICATIONS FROM CROSS-CULTURAL STUDIES

**DOI:** 10.1093/geroni/igz038.3012

**Published:** 2019-11-08

**Authors:** Nan Jiang, Jing Wang

**Affiliations:** 1 Columbia University, New York, United States; 2 Duke University School of Nursing, Durham, North Carolina, United States

## Abstract

The impact of social determinants on health is well documented. Among them, social support has emerged as one of the most important old ideas being revisited from a new perspective. Social support refers to perceived supportive resources from an individual’s social engagement or social network. Recently, the concept of social support has become the subject of intense discussion, as it represents a mechanism by which interventions lead to health improvement. Neuropsychological disorders, on the other hand, represent a large burden on worldwide health. Research has shown that older adults with neurological conditions are embedded in social structures that may affect their outcomes, but not enough attention has been paid to the potential effect of social support on many neurological conditions. Using data collected from studies across cultures that have sought to understand neurological well-being worldwide, this symposium will present evidence of the relationship between social support and neurological conditions among older adults and explore how these mechanisms of social support extend the understanding of health disparities in old-age neurological wellbeing. A discussant will draw out common themes from these papers and connect them with the broader literature on the effect of social support on neurological well-being. We propose that social support constitutes an integral part of medical care for older adults with neurological conditions. This symposium will generate insights to help clinical practitioners more effectively design their social support interventions.

